# Foot–ankle therapeutic exercise program can improve gait speed in people with diabetic neuropathy: a randomized controlled trial

**DOI:** 10.1038/s41598-022-11745-0

**Published:** 2022-05-09

**Authors:** Renan L. Monteiro, Jane S. S. P. Ferreira, Érica Q. Silva, Ronaldo H. Cruvinel-Júnior, Jady L. Veríssimo, Sicco A. Bus, Isabel C. N. Sacco

**Affiliations:** 1 Physical Therapy, Speech and Occupational Therapy Department, Faculdade de Medicina, Universidade de São Paulo, Rua Cipotânea, 51-Cidade Universitária, São Paulo, São Paulo 05360-160 Brazil; 2 Department of Biological Science and Health, Federal University of Amapá, Macapá, Brazil; 3 Department of Rehabilitation Medicine, Amsterdam Movement Sciences, Amsterdam UMC, University of Amsterdam, Amsterdam, The Netherlands

**Keywords:** Health care, Health occupations

## Abstract

This study sought to determine whether a foot–ankle therapeutic exercise program can improve daily physical activity (i.e. number of steps) and fast and self-selected gait speed in people with diabetic peripheral neuropathy (DPN). In this single-blind randomized controlled trial and intention-to-treat analysis, 78 volunteers with DPN were allocated into a control group, which received usual care, and an intervention group (IG), which received usual care plus a 12-week foot–ankle exercise program. The adherence at 12 weeks rate in the IG was 92.3% (36 participants) and the dropout was 5.1% in the control group (2 participants). The number of steps and self-selected gait speed did not change significantly in either group (p > 0.05), although a 1,365-step difference between groups were observed at 1-year followup. The 12-week foot–ankle therapeutic exercises improved significantly fast-gait speed (primary outcome) (p = 0.020), ankle range of motion (p = 0.048), and vibration perception (secondary outcomes) (p = 0.030), compared with usual-care at 12 weeks. At 24 weeks, the IG showed better quality of life than controls (p = 0.048). At 1-year, fast-gait speed and vibration perception remained higher in the IG versus controls. Overall, the program may be a complementary treatment strategy for improving musculoskeletal and functional deficits related to DPN.

**Trial registration** ClinicalTrials.gov NCT02790931 (06/06/2016).

## Introduction

Diabetic peripheral neuropathy (DPN), an important risk factor for amputation and reduced physical mobility, occurs in more than 50% of people with diabetes^1^. DPN is associated with decreased muscle strength and physical activity level, as measured by steps per day^2^ and reduced gait speed^3^. Daily steps in persons with DPN (PWDPN) are inversely proportional to the amount of intramuscular adipose tissue^4^, suggesting that muscle impairment is a factor underlying decreased physical activity. Other studies suggest that motor and sensory deficits^2,5^ and reduced foot–ankle range of motion (ROM)^3,6^ are directly related to decreased physical activity levels, as are the reduced quality of life (QoL) and decreased gait speed associated with DPN^5^.

Physical functionality, a third WHO health indicator alongside mortality and morbidity, requires prioritizing rehabilitation and prevention of musculoskeletal disorders^7^. Compiled data from *Global Burden of Diseases, Injuries and Risk Factors* (1990 and 2019), considering 25 health conditions that could benefit from rehabilitation, indicated that, in terms of prevalence and years of life lived with disability, the top condition for almost 30 years has been musculoskeletal disorders^7^; one in every three people worldwide would benefit from rehabilitation. Diabetes progression and DPN compromise musculoskeletal function, leading to limitations in everyday physical functioning. Furthermore, according to the WHO (2021), diabetes prevalence has been rising more rapidly in low- and middle-income countries, in Brazil for instance, than in high-income countries, and this unequal advance is coupled with a scarcity of studies that focus on rehabilitation in this population. Thus, there is a strong need for further investigations of rehabilitation strategies for musculoskeletal conditions worldwide, especially related to motor dysfunctions resulting from diabetes and DPN progression.

Controlled and non-controlled studies have sought to assess the effects of different exercise therapy strategies, including foot-related exercises, balance training, and weightbearing and resistance exercises, on different DPN-related outcomes^8^. These findings provided the foundation for the International Working Group on Diabetic Foot (IWGDF; 2020) rehabilitation strategy recommendations, such as foot- and mobility-targeted exercises, to mitigate risk factors for foot ulceration. However, while they showed that these exercises may improve DPN symptoms and increase ankle-joint ROM, it is still unclear whether they could improve foot–ankle muscle strength and functionality in people with a low or moderate risk of foot ulceration^8^. In addition, the evidence is still weak because the majority of randomized controlled trials (RCTs) addressing this are of low quality, present small effect sizes, and do not involve exercises that specifically target the main musculoskeletal dysfunction in PWDPN. Further, the variety of described foot-related exercises preclude definitive conclusions about their effectiveness^8^. The innovation and relevance of this study is based on: (1) the development of a specific-exercise protocol focusing on DPN-related musculoskeletal deficits; (2) with a group-based exercise rehabilitation program; (3) a robust study design, including primary outcomes that reflect the patient's physical functioning and quality of life; (4) aiming to improve the certainty of evidence about the effects of foot–ankle exercise in PWDPN.

The primary aim of this RCT was to investigate the effects of a 12-week foot–ankle therapeutic exercise program on daily physical activity level (number of steps measure by an accelerometer) and self-selected and fast-gait speeds in PWDPN. The secondary aims were to investigate the effectiveness of this intervention at 6, 12, and 24 weeks on passive and static ankle-joint ROM, tactile (10-g monofilament) and vibration sensitivity (tuning fork), DPN symptoms (Michigan Neuropathy Screening Instrument), QoL by the EQ-5D questionnaire, foot health and functionality by the Foot Health Status Questionnaire, hallux and toe muscle strength (pressure platform), and foot ulcer incidence at 1-year followup. Originally, all primary and secondary outcomes were planned to be assessed at 1-year followup; however, due to the COVID-19 pandemic, these aims were modified^9^.

## Results and discussion

**Figure 1:**
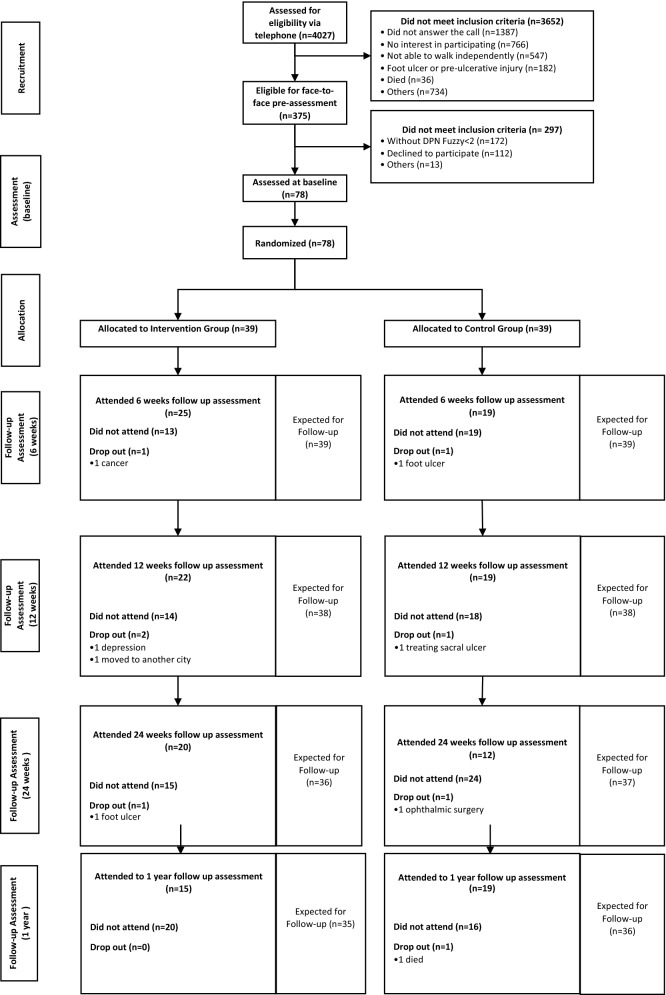
Flowchart of recruitment, assessment, and follow-up process.

Baseline assessment data are described in Table 1. In the IG, 36 participants (92.3%) completed the 12-week exercise program (Fig. 1). The dropout rate at 12 weeks was 5.1% in the CG (2 participants); reasons for dropout in both groups are described in Fig. 1. The dropout at 24 weeks included an additional participant in each group (2.6%). After 1 year, only one participant, in the CG, dropped out (2.6%). Absence was high for the 6-, 12- and 24-week and 1-year followup visits due to the COVID-19 pandemic (Fig. 1). Therefore, mitigating strategies to improve internal and external study validity were adopted to alter the originally planned methods and statistical analysis.

**Table 1:** Control and intervention group characteristics at baseline.

Variables	Intervention group (n = 39) Mean (SD)	Control group (n = 39) Mean (SD)	p-value	95% (CI) for mean estimated difference
Age (years)	61.5 (11.7)	60.1 (8.9)	0.259	
Height (m)	1.6 (0.08)	1.6 (0.09)	0.490	
Body mass (kg)	77.3 (14.0)	80.8 (16.4)	0.145	
Daily physical activity (number of steps)	8.092 (4.230)	7.641 (4.087)	0.599	[− 1.321, 2.300]
Fast gait speed (m/s)	1.5 (0.2)	1.5 (0.3)	0.881	[− 0.08, 0.19]
Self-selected gait speed (m/s)	1.1 (0.2)	1.0 (0.1)	0.264	[− 0.04, − 0.14]
MNSI (score)	6.3 (2.9)	6.3 (1.9)	0.779	[− 0.7, 1.2]
FHSQ—foot pain (score)	55.8 (28.3)	55.3 (26.0)	0.930	[− 11.6, 12.1]
FHSQ—foot function (score)	70.3 (26.5)	64.2 (26.9)	0.320	[− 0.5, 22.8]
FHSQ—shoes (score)	49.3 (37.0)	44.4 (35.6)	0.546	[− 10.8, 21.4]
FHSQ—foot health (score)	26.7 (23.3)	33.2 (27.8)	0.274	[− 15.2, 8.4]
Ankle plantaflexion ROM L (◦)	31.8 (7.2)	31.8 (7.9)	0.989	[− 4.5, 1.5]
Ankle plantaflexion ROM R (◦)	28.7 (8.8)	29.6 (8.3)	0.598	[− 4.9, 1.8]
Ankle dorsiflexion ROM L (◦)	18.4 (6.7)	19.3 (7.4)	0.207	[− 4.85, − 0.01]
Ankle dorsiflexion ROM R (◦)	17.2 (6.5)	17.8 (6.2)	0.589	[− 4.4, 0.8]
Tactile sensitivity (number of areas)	2.2 (2.3)	2.5 (2.5)	0.130	[− 2.0, 0.2]
Tactile-Threshold-L	3.8 (1.4)	4.0 (1.7)	0.596	[− 0.9, 0.5]
Tactile-Threshold-R	3.9 (1.4)	3.5 (1.5)	0.422	[− 0.4, 0.9]
Vibration—L	1.6 (0.8)	1.5 (0.7)	0.662	[− 0.2, 0.4]
Vibration—R	1.5 (0.8)	1.4 (0.7)	0.394	[− 0.1, 0.6]
Quality of life (score)	0.59 (0.1)	0.59 (0.2)	0.905	[− 0.05, 0.10]
Hallux strength—(%BW)	12.1 (6.3)	12.2 (4.9)	0.911	[− 2.6, 2.3]
Toe strength—(%BW)	7.9 (5.1)	8.3 (4.4)	0.676	[− 2.1, 2.1]

*MNSI* Michigan Neuropathy Screening Instrument, *FHSQ* Foot Health Status Questionnaire, *ROM* range of motion, *L* left, *R* right, *BW* body weight.

**Figure 2:**
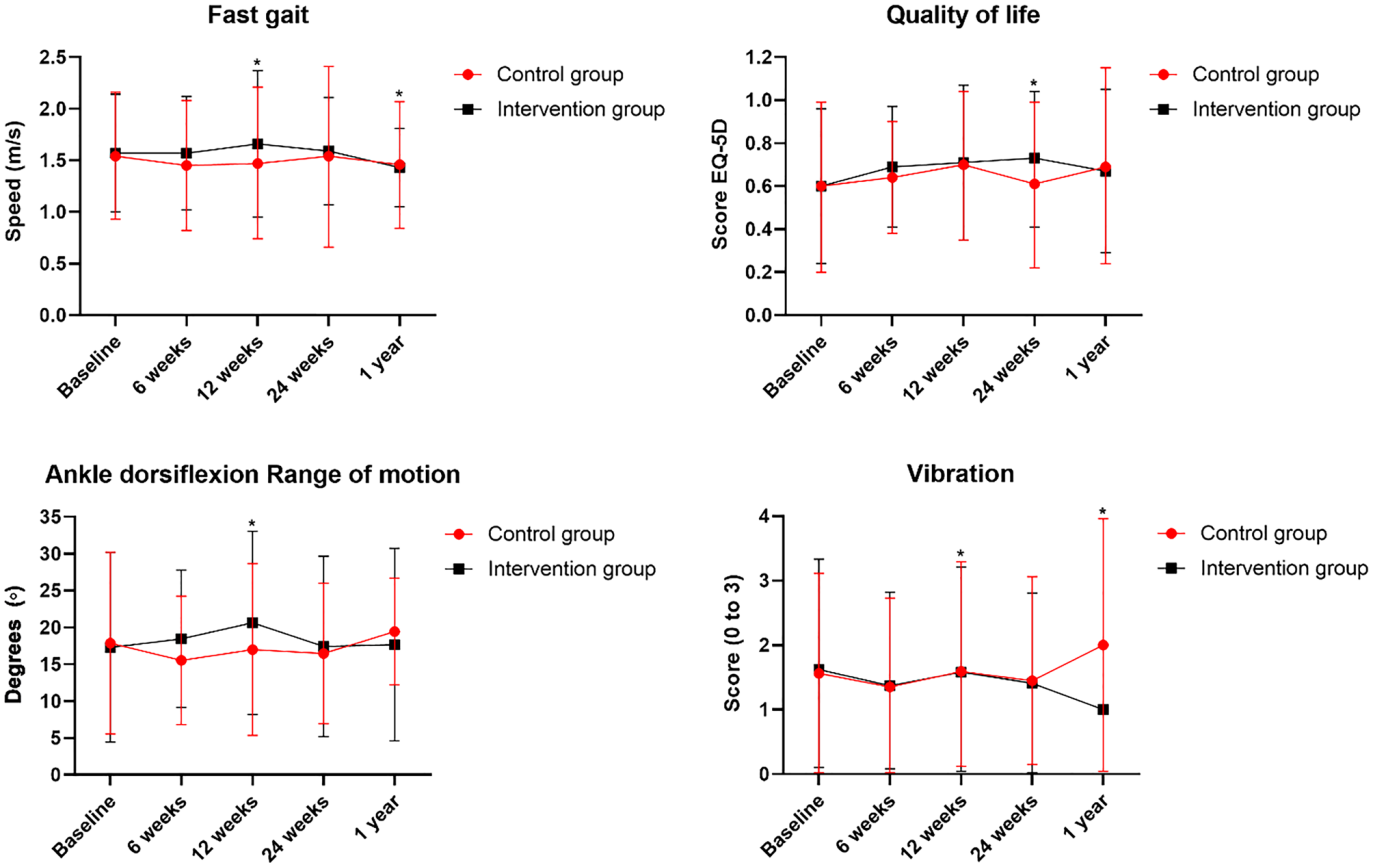
Different between intervention group and control group on fast gait speed, quality of life, ankle range of motion and vibration outcomes.

According to the IWGDF, a major focus in the prevention of plantar ulcers is treatment of modifiable risk factors^8^. Our study aimed to evaluate the effectiveness of foot–ankle exercise training on lower limb function and on modifiable risk-factor outcomes in PWDPN. The results (Table 2, Fig. 2, and Table 2 in the “Supplementary material”) and discussion presentations are organized and structured as patient, intervention, comparison, outcome (PICO) questions for each modifiable risk factor evaluated in this RCT.

**Table 2:**
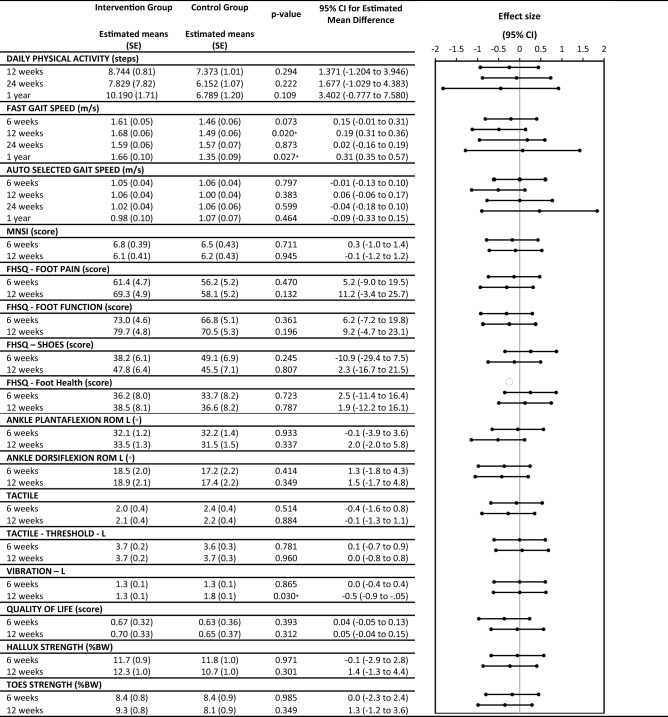
Secondary and primary outcomes from intervention group and control groups.

### In PWDPN, can the addition of a foot–ankle therapeutic exercise program improve gait speed?

Fast-gait speed, but not self-selected gait speed, was significantly affected by the foot–ankle exercise program compared with usual care alone (Table 2, Fig. 2). After 12 weeks, IG participants walked faster than CG participants (p = 0.020; interaction effect), and after 24 weeks and 1 year, the IG still maintained significantly greater fast-gait speed (Table 2, p = 0.027; interaction effect).

DPN leads to a deterioration of lower limb motor function, with rapid decrements of ankle strength^10^ and intrinsic foot muscle strength and size^11,12^. Decreased muscle strength is directly related to worsening of functional abilities such as balance, walking, and gait speed^13^. Slower gait speed is related to increased stride time variability, which increases the risk of falls in the elderly^14^, and reduced gait speed is also independently associated with greater risk of mortality in older adults^15^. Moreover, White et al.^16^ reported an association between decreased gait speed and increased risk of death among older adults. Thus, it is clear that gait speed is closely linked to general health-related outcomes in older adults, as the participants of this RCT. Furthermore, reduced gait speed in PWDPN is related to low levels of physical activity^3^, and thereby increases the risk of developing ulcers^17^. The exercise program did not affect self-selected gait speed, another relevant outcome for PWDPN, perhaps due to its lower sensitivity to change. Taveggia et al.^18^ also observed significant improvement in fast but not self-selected gait speed after exercise-based multimodal treatment in PWDPN.

IG participants not only increased their fast-gait speed after 12 weeks of the program; they also maintained this greater speed, even after 1 year. An improvement of 0.10 m/s in usual walking speed predicts a substantial reduction in mortality in older adults^19^ and an increase of 0.11 m/s in fast-gait speed in persons with musculoskeletal dysfunctions, such as DPN, is considered clinically important^20^. In our study, IG participants showed a mean difference of 0.18 m/s compared with CG participants, a greater increase than that recommended for clinical improvement and mortality reduction. Thus, our foot–ankle exercise program potentially promotes an indirect protective effect against mortality risk.

Melese et al.^21^ reviewed the effectiveness of different exercise modalities on gait speed in DPN subjects, including five studies on various exercise modalities, such as gait and balance training, proprioceptive training, ROM exercises, and lower limb strengthening. Only two studies included foot–ankle specific training resembling ours in their protocols^21^; both observed an increase in self-selected speed over time in the experimental group. To date, we are not aware of any previous study investigating the effectiveness of foot–ankle training on fast-gait speed.

Although our program focused mainly on gaining muscle strength and ROM, the functional exercises in the protocol could have also induced faster walking speed. The gains in fast-gait speed achieved could improve lifestyle via metabolic control and health benefits, especially in participants with poor aerobic resistance and overweight, who might find it difficult to engage in moderate to intense daily-living activities^22,23^. Faster gait speed ability may help improve functional capacity for aerobic activity, including daily living activities involving motor challenges, such as rushing to catch a bus, cross a street, or be on time for an appointment.

### In PWDPN, can the addition of a foot–ankle therapeutic exercise program improve daily physical activity levels?

Considering that foot exercises were able to improve outcomes related to daily performance, we hypothesized that the number of steps participants take in their daily living activities would also be improved after the intervention. The number of steps taken over a 6-day period did not differ between groups after 12 weeks of foot–ankle exercises (Table 2 and Fig. 2). This may be due to step count not being sensitive enough; a search for other outcomes that represent daily activity may be needed. A closer look at this negative result, however, revealed that while all participants started the study at a moderate activity level (7641 and 8092 steps in the CG and IG, respectively), the CG showed steadily decreasing activity, to a low activity level (7093 steps) by the 1-year followup, according to Tudor-Locke and Bassett (2004) classification. The IG, in contrast, remained at a moderate activity level at the 1-year followup (8458 steps).

Steps are a fundamental unit of human locomotion, and thus are a preferred metric for quantifying physical activity^24^. The status of being moderately active represented a health advantage for people in the IG, because in addition to helping with lifestyle and daily living activity, more steps could improve musculoskeletal capacity, especially foot–ankle muscle strength^25^. This could improve the performance of daily locomotor tasks, and also benefit metabolic and glycemic control^26^.

Some bias may have been introduced in that steps per week, although measured with an accelerometer, are to a certain extent self-reported, because participants themselves read and recorded the number of daily steps displayed on the accelerometer. Some participants reported that they occasionally forgot to write down their steps, a possible bias also noted and discussed by other authors^27^. A systematic review concluded that self-report measures of physical activity can be both higher and lower than directly measured physical activity levels^28^, suggesting variability in self-report measures.

The IWGDF^29^, recommends informing persons with diabetes at low or moderate risk for foot ulceration (IWGDF risk 1 or 2) that a moderate increase in daily walking-related weightbearing activity (e.g., an extra 1000 steps/day) is safe and does not increase ulcer risk. Although neither group increased step number by 1000 during the study, the IG increased step number by approximately 400 after 1 year, which is still within a safe increase according to the IWGDF. The CG decreased step number by approximately 600 after 1 year. This difference, while not significant, is notable; a larger sample size may shed more light on this.

IWGDF guidelines also focus on risk factors for ulceration, and recommend foot-related exercises as a prevention strategy^29^, but RCTs on foot–ankle exercises for PWDPN are still scarce. To date, we are not aware of any study investigating the effects of exercises targeted specifically to the foot–ankle complex on daily physical activity. Grewal et al.^30^ and Mueller et al.^31^ showed increases in step number in PWDPN after foot-related exercise; however, their programs included general balance or weightbearing exercises, rather than foot–ankle specific exercises, as in our program. Therefore, although they are recommended, evidence regarding foot–ankle exercises and physical activity levels in PWDPN is still weak.

### In PWDPN, can the addition of a foot–ankle therapeutic exercise program improve toe strength and ankle-joint ROM?

As outcomes related to musculoskeletal function, such as muscle strength and joint ROM, are of paramount importance for PWDPN, we sought to also assess the effectiveness of our foot–ankle exercise program on ankle ROM and muscle strength of the toes (including hallux) in PWDPN. After 12 weeks of foot–ankle training, the IG showed increased ankle dorsiflexion ROM compared with the CG (p = 0.048; interaction effect, Table 2, and Fig. 2). In the 24-week and 1-year followups, there were no differences between groups in the ROM (Table 2 in the “Supplementary material”). In addition, there were no significant differences between foot–ankle training and usual care on toe muscle strength (Table 2, and Table 2 in the “Supplementary material”).

A systematic review assessing the effects of foot- and mobility-related exercises on foot–ankle muscle strength in PWDPN concluded that their efficacy is still unclear^8^. Out of the three studies included in the systematic review^25,32,33^, two showed increased foot–ankle strength and one found no effect^34^. It is important to highlight the heterogeneity of the methods used to assess foot–ankle muscle strength, which hinders the ability to analyze the efficacy of foot-related exercises for this outcome.

Regarding foot–ankle ROM, a cross-sectional study of 281 individuals revealed that people with or without DPN experienced limited joint mobility in all foot joints^35^. According to a systematic review by Monteiro-Soares et al.^36^, limited subtarsal and first metatarsophalangeal joint mobilities were associated with diabetic foot ulcer development. For this reason, this modifiable risk factor was targeted in our intervention, and has been a common target in other foot-related exercise interventions focusing on foot-health and musculoskeletal improvement in PWDPN^25,37,38^. Our study showed improved ankle dorsiflexion ROM in the IG compared with the CG after 12 weeks of foot–ankle training. Our results corroborate other investigations that found an increase in ankle dorsiflexion ROM after 4 weeks of foot-targeted exercises^37^ and an increase in ROM of the first metatarsophalangeal joint after an 8-week foot-targeted exercise program^38^. Only one RCT found no differences in ROM of the ankle and first metatarsophalangeal joints after a 12-week foot-targeted exercise program^25^. A noteworthy difference between the studies was that Cerrahoglu et al.^37^ and Kanchanasamut et al.^38^ applied general and balance exercises in their protocols in addition to the foot-targeted exercises, unlike Sartor et al.^25^, who focused specifically on foot–ankle exercises. The addition of these general and balance exercises probably helped to improve foot–ankle ROM in the PWDPN. Our protocol included functional exercises as well, which may also have contributed to the improvement in ankle dorsiflexion ROM after 12 weeks of exercise.

Improvements in foot–ankle ROM should indirectly lead to better locomotor performance and more independence and autonomy for PWDPN regarding daily-living activities. Therefore, our findings and the positive results from other cited RCTs and non-controlled studies^32,39,40^ reinforce the importance of exercising the foot–ankle to gain this clinically relevant outcome^36^. Furthermore, foot- and mobility-related exercises may be beneficial for improving other modifiable risk factors for foot ulceration, such as foot sensitivity and DPN symptoms^8^.

### In PWDPN, can the addition of a foot–ankle therapeutic exercise program improve DPN symptoms and tactile and vibration sensitivities?

The foot–ankle intervention did not affect DPN symptoms and tactile sensitivities (Table 2, and Table 2 in the “Supplementary material”). According to van Netten et al.^8^, evidence that foot and mobility-targeted exercises may improve DPN symptoms is low-quality due to inconsistency and imprecision of study design, with small effect sizes and large confidence intervals.

The clinical importance of vibration sensitivity for the development of diabetic foot ulcers has been demonstrated by research associating current or past diabetic foot ulcers with altered tuning fork vibration perception^36^. In addition, Zippenfennig et al.^41^ reported worse vibration perception thresholds in PWDPN compared with controls and people without DPN. In our study, after 12 weeks of foot–ankle training, the IG presented better vibration sensitivity compared with the CG (p = 0.030; interaction effect, Table 2, and Fig. 2), and that difference was maintained at the 1-year followup assessment (p = 0.023; interaction effect, Table 2 in the “Supplementary material”).

Aerobic exercise may activate increased Schwann cell proliferation, a phenomenon that may play an important role in stimulating axonal regeneration^42^. It is possible that our foot–ankle exercise protocol provided sufficient stimulation to achieve such a cellular effect. This exercise-induced increase in peripheral nerve regeneration has been shown to promote improvements in both functional and morphological markers of nerve and motor function in mice^43^. Furthermore, a supervised aerobic exercise program performed 4 h per week (brisk walking on a treadmill) was able to significantly improve vibration perception thresholds in people with diabetes over a 4-year period^44^. These axonal responses and sensory and motor improvements might be the reason for the benefits in vibration perception and functional performance, such as the increase in fast-gait speed, that we observed in the IG participants. Whereas most studies on peripheral sensory function have evaluated the effects of aerobic exercise, our results revealed that exercise focusing on the foot–ankle joints can also be beneficial, indicating the promise of such exercise as a complementary treatment for prevention of complications from DPN.

### In PWDPN, can the addition of a foot–ankle therapeutic exercise program improve QoL, foot health, and functionality?

Because we observed changes in locomotor function (fast-gait speed), joint ROM, and vibration sensitivity, we speculated that these gains together would also improve QoL and functionality in PWDPN. The foot–ankle exercise program yielded a positive effect on QoL at the 24-week followup compared with CG (p = 0.048; interaction effect, Table 2 in the “Supplementary material”). Compared with baseline, the IG showed a significantly improved QoL score at 12 (p = 0.006, time effect, Table 2 and Fig. 2) and 24 (p = 0.006, time effect, Table 2 in the “Supplementary material”) weeks.

Exercise can improve QoL through improving DPN symptoms^25^, foot–ankle ROM^33,37,38^, functionality^25^, muscle strength^32,33^, and foot rollover^25^. Aerobic exercise, resistance exercise, combined exercise, and yoga all have a positive effect on QoL compared with usual care in people with type 2 Diabetes^45^. A pretest–posttest study with a nonequivalent control group assessing the effects of a Tai Chi Chuan program in diabetic patients^46^ found improvement in different domains of the Korean SF-36 questionnaire. Although few studies have evaluated the effect of specific foot–ankle exercises on the QoL of PWDPN, self-care associated with exercise practice has been shown to lead to a better QoL in people with diabetes^47^. Our program resulted in improved QoL in the IG that was manifested after 12 and 24 weeks.

Also, after 12 and 24 weeks, the IG participants improved their foot pain scores compared with baseline assessment (p = 0.044 and p = 0.026; time effect, respectively). The CG also showed improvements in foot health after 1 year, compared with baseline and 6 weeks (p = 0.001 and p = 0.025; time effect, respectively). The foot-health improvement in the CG might be due to the usual-care guidance offered to the patient during the orientation session. This could have been sufficient to improve foot functionality, as revealed by the FHSQ scores. The placebo effect is an important factor to be considered, especially in physical therapy trials. The patient-physiotherapist relationship involves warmth, confidence, friendliness, support, sympathy, language reciprocity, use of psychosocial talk, eye contact, smiling and caring expressions of support and interest, and interpretation of the patient's nonverbal cues and expressions, and this relationship is established alongside a treatment regimen. All of these serve to potentiate placebo effects^48^.

### In PWDPN, can the addition of a foot–ankle therapeutic exercise program better prevent foot ulceration?

Over a 1-year followup, only two participants developed a plantar foot ulcer, one from the IG and one from the CG. The IG participant was diagnosed approximately 13 weeks after randomization, whereas the CG participant was diagnosed approximately 5 weeks after randomization. Due to an insufficient number of participants with foot ulcers, we cannot say whether the later time to develop an ulcer in the IG participant was linked to the intervention.

## Strengths and limitations

The strengths of this study include the rigorous RCT methodology and adoption of a robust statistical model (GMM), a larger sample size than other studies in the same field^25,37,38^, and a group intervention approach with individual progression that integrates incremental gains. One limitation was a relatively high dropout rate during followup visits, mainly due to the COVID-19 pandemic. Considering that we did not quantify the amount of load/intensity of each exercise, mainly due to the nature of the functional exercises in the physical therapy practice for older adults, an insufficient workload among some participants may be a reason for the absence of effects in some variables, such as foot muscle strength. However, we opt to follow the protocol previously published for study reproducibility reasons to other researchers and rehabilitation studies. Furthermore, other parameters related to the clinical control of diabetes, such as glycated hemoglobin and glycaemia, were not assessed, and might have influenced our functional and clinical outcomes.

We believe that the improvements seen in the IG participants in several functional outcomes, such as foot–ankle ROM and fast-gait speed, as well as clinical outcomes such as vibration sensitivity, had a direct impact on general clinical improvement in the IG, as evidenced by increased QoL and foot-health measures. We suggest future mediation analysis of our clinical trial data to further understand which outcomes indirectly influenced the changes observed in QoL in intervention participants. We planned and conducted an interim analysis that was published as a feasibility study^49^, but its outcomes did not drive our choices of mitigating strategies for responding to extenuating circumstances. The main purpose of the planned interim data analysis was to analyze recruitment and adherence rates and potential changes in the outcomes, and not to plan for mitigating strategy implementations.

## Conclusions

We conclude that the 12 weeks of the foot–ankle therapeutic exercise program showed positive effects compared with usual care on the primary outcome of fast-gait speed, and on the secondary outcomes of foot–ankle ROM, vibration sensitivity, and QoL. However, no effects were seen on the two other primary outcomes after 12 weeks (self-selected gait speed and number of steps), although a 1365-step difference between groups were observed at 1-year followup. Improvements in vibration sensitivity and ROM may indicate an improvement in modifiable risk factors for foot ulceration, whereas an increase in gait speed may be an indicator related to mortality reduction in this population. Taken together, the findings of our study suggest that foot–ankle exercises may be an effective complementary treatment strategy for improving some musculoskeletal and functional deficits related to DPN. For other outcomes, larger trials are needed to further investigate the effects of such an exercise program.

## Methods

### Design

A 12-month, single-blind, parallel-group, two-armed superiority RCT, prospectively registered at ClinicalTrials.gov (NCT02790931; 06/06/2016), under the name “Effects of foot muscle strengthening in daily activity in diabetic neuropathic patients”, was designed following CONSORT recommendations. The protocol is published elsewhere^50^. All methods were carried out in accordance to the Resolution 196/96 of the National Health Council and the trial was approved by the Ethics Committee of the School of Medicine of the University of São Paulo (Research protocol No. 1.464.870, approved on 24/03/2016).

Unfortunately, we faced an extenuating circumstance during the trial development. The most impactful modifications made in the methods and statistical analyses were reported accordingly to the parties responsible for planning, reviewing, and approving the study; namely, all the study staff, institutional ethics committee, data-monitoring committee, Institutional graduate studies committee, and the research funding agency (São Paulo Research Foundation—FAPESP, https://fapesp.br/en).

### Participants and recruitment

Participants signed an informed consent form approved by the Ethics Committee of the School of Medicine of the University of São Paulo (24/03/2016, protocol No. 1.464.870). The main researcher explained to each eligible participant every step of the assessment and follow-up, possible risks, and that no compensation or benefits were to be expected. There was no patient and public involvement in this study.

Sample size calculation was based on three outcomes: daily physical activity (number of steps), and self-selected and fast-gait speeds. The following parameters were used in GPower v. 3.1^51^: a statistical design of F-test repeated measures and interaction between and within factors with 3 repeated measures (baseline, 12 weeks, and 1-year followup) and two study groups (control and intervention); a statistical power of 0.80; an alpha of 0.05; and effect sizes of 0.175, 0.170, and 0.154 for fast-gait speed^52^, self-selected gait speed^53^ and number of steps^30^, respectively. The calculated sample sizes were 54, 58, and 70, respectively. Thus, the sample size was based on the number of steps, which required the largest number of participants (n = 70). Assuming a 10% total dropout rate, we recruited 78 participants between December 2017 and December 2019 using digital social media advertising, outpatient clinic databases, and direct contact with people with diabetes during health campaigns at the university. Eligibility criteria included both sexes, age between 18 and 75 years; type 1 or 2 diabetes mellitus with moderate DPN as diagnosed by a fuzzy decision support system^54^; ability to walk independently for at least 10 m; a maximum of one amputated toe, which could not be the hallux; and access to electronic devices with internet allowing usage of our web-software. The exclusion criteria were: presence of an active plantar ulcer; history of surgical procedure at the hip, knee, or ankle, or indication of surgery throughout the intervention period; history of arthroplasty and/or current use of orthosis for the lower limbs, or indication of lower limb arthroplasty throughout the intervention period; diagnosis of neurological disease; dementia or inability to give consistent information; undergoing any physiotherapy care during the intervention period; and major vascular complications and/or severe retinopathy.

The allocation to the intervention group (IG) or control group (CG) was blind, based on a numerical code sequence prepared by an independent researcher (Clinstat software, University of York, York, UK). The allocation sequence was kept in opaque, sequentially numbered envelopes. All baseline and followup assessments were performed by physiotherapists blinded to the treatment allocation. Participants’ data were kept confidential throughout the study by encoding their names. The trial statistician was blinded to treatment allocation until the main analysis had been completed. A flowchart summarizing the clinical trial procedures is shown in Fig. 1.

### Treatment arms

CG participants received the usual care recommended by medical staff and by the guidelines of the IWGDF^29^ at the baseline session (Fig. 1).

IG participants received the usual care (inspect your feet daily, wear socks without elastic and seams, cut your nails properly, avoid cutting corns or blisters without supervision, avoid going barefoot or wearing shoes without socks or slippers, and seek medical attention whenever you identify foot problems), along with a 12-week foot–ankle exercise program. The exercise protocol was performed twice weekly under in-person supervision by a physiotherapist, and twice weekly at home, remotely supervised through Educational Diabetic Foot Software (SOPeD, www.soped.com.br). Both protocols (SOPeD and in-person supervised foot–ankle exercises) consisted of the same set of modules: (a) warm-up exercises, (b) intrinsic foot muscle strengthening, (c) extrinsic foot–ankle muscle strengthening, and (d) functional exercises, such as balance and gait training. The in-person supervised sessions were conducted in groups of five to eight participants. To gain muscle strength, it is mandatory to manage exerciss intensity by manipulating the parameters of the training program according to the individual’s needs, such as number of repetitions and sets^55^. Therefore, the present protocol included exercises that could be increased in intensity individually by the physiotherapist when the participant was able to perform the exercise correctly, which ranged from 1 to 3 sets, 5–40 repetitions, when not referring to any pain or cramps, as any other face-to-face rehabilitation program. The exercises performed at home, using the software, were progressed based on an algorithm that adjusts the training volume based on the perceived exertion assessment and reported by the user through a visual analogue scale. The complete exercise program is published elsewhere^50^ and a general description is in the supplementary file (“Supplementary material”, Table 1).

### Assessments

The assessments consisted of five evaluations: baseline, 6 weeks, 12 weeks, 24 weeks, and 1-year followup, performed by evaluators who were blinded to group allocation. The daily physical activity level was measured by counting the number of steps using a 3D accelerometer, for six consecutive days (Power Walker-610, Yamax, Japan). For self-selected and fast-gait speeds, two photocells (CEFISE, Speed Test Fit Model, Nova Odessa, Brazil) located in the middle (at the 6-m mark) of the 10-m walkway were used. For both speeds, 3 trials were performed and the mean was used for statistical purposes. The number of plantar areas in which the participant did not feel pressure applied using a 10 g monofilament was recorded as the tactile sensitivity^56^. The tactile sensory threshold was assessed on the dorsal surface of the hallux, according to Jeng et al.^57^. Vibration sensitivity was assessed by the timed method using a 128 Hz tuning fork applied to the dorsal surface of the distal phalanx of the hallux on both feet^58^. Passive ankle ROM was assessed bilaterally by an ankle electrogoniometer (model SG110/A, Biometrics, Gwent, UK) with the participant in supine position. Hallux and toe strength were assessed by an emed-q pressure platform (Novel, Munich, Germany), as described previously^59^.

Originally, the number of steps, gait speed, sensitivities, ankle ROM, and foot strength were planned to be assessed at each of the five assessment time points. However, due to the COVID-19 pandemic, the 24-week and 1-year followup assessments could not be performed^9^.

DPN symptoms were evaluated by the Brazilian version of the Michigan Neuropathy Screening Instrument (MNSI)^60^. QoL was assessed by the EuroQoL 5-dimensions (EQ-5D) questionnaire^61^. Foot health and functionality were assessed by the Brazilian-Portuguese version of the FHSQ-BR, a foot-health status questionnaire^62^, with scores calculated using FHSQ software, version 1.03 (Care Quest, Australia). Originally, the MNSI, FHSQ-BR, and EQ-5D were planned to be administered in-person at each assessment, at the laboratory. However, due to the COVID-19 pandemic, the 24-week and 1-year followup assessments were conducted by telephone^9^.

Plantar foot ulcers were also assessed throughout the study (12-month period). If an ulcer occurred during either the intervention or the followup periods, a nurse specialist in diabetic foot with 14 years of experience assessed photographs of the ulcer and determined whether the occurrence was indeed an ulcer. A diabetic foot ulcer was defined as a “full thickness lesion of the skin distal to the malleoli in a person with diabetes mellitus”^63^. When a participant developed a plantar foot ulcer, the intervention was discontinued, but the subject was still included in the intention-to-treat analysis.

### Statistical analysis

Statistical analyses were performed using the *Statistical Package for the Social Sciences* (SPSS, IBM; v.23.0), adopting a 5% significance level. All analyses used the full set of randomly assigned participants under the intention-to-treat assumption. Originally, the statistical analysis was planned to be performed using ANOVAs; however, due to the COVID-19 pandemic and the consequent large amount of missing data, a Generalized Linear Mixed Model (GLMM) method was adopted^9^. Analysis determined that the missing data could be considered to be missing completely at random. The GLMM method was then used for univariate analyses, considering the following as factors: groups (CG and IG), time of assessment (baseline, 6 weeks, 12 weeks, 24 weeks, and 1 year), and the interaction effect (time by group), which was our primary outcome comparison. Participants and time were considered as random effects and groups as fixed effects in the GLMM modeling. Q-Q graphs were plotted to verify the adequacy (normality) of each model. Univariate (main effects) and multivariate (interaction effect) comparisons of the estimated marginal means were adjusted with the Bonferroni correction. Comparisons between pairs of estimated marginal means were made based on the original scale of each of the dependent variables of the study.

## Supplementary Information

Supplementary Tables.

## Data Availability

Data are owned by the Laboratório de Biomecânica do Movimento e Postura Humana—LaBiMPH, Departamento de Fisioterapia, Fonoaudiologia e Terapia Ocupacional, Faculdade de Medicina da Universidade de São Paulo. Request to use, share and disseminate such data must be sent to icnsacco@usp.br.
