# Author Correction: Foot–ankle therapeutic exercise program can improve gait speed in people with diabetic neuropathy: a randomized controlled trial

**DOI:** 10.1038/s41598-022-16172-9

**Published:** 2022-07-11

**Authors:** Renan L. Monteiro, Jane S. S. P. Ferreira, Érica Q. Silva, Ronaldo H. Cruvinel-Júnior, Jady L. Veríssimo, Sicco A. Bus, Isabel C. N. Sacco

**Affiliations:** 1 Physical Therapy, Speech and Occupational Therapy Department, Faculdade de Medicina, Universidade de São Paulo, Rua Cipotânea, 51-Cidade Universitária, São Paulo, São Paulo 05360-160 Brazil; 2 Department of Biological Science and Health, Federal University of Amapá, Macapá, Brazil; 3 Department of Rehabilitation Medicine, Amsterdam Movement Sciences, Amsterdam UMC, University of Amsterdam, Amsterdam, The Netherlands

Correction to: *Scientific Reports* 10.1038/s41598-022-11745-0, published online 09 May 2022

The original version of this Article contained errors in Table 2. In the columns ‘Intervention Group’ and ‘Control Group’, the mean and standard deviation values were reported, instead of the estimated mean and standard error values. The original Table 2 and accompanying legend appear below. As a result, in the Supplementary Tables file, Table 1 contained the same errors.

**Table 2:**
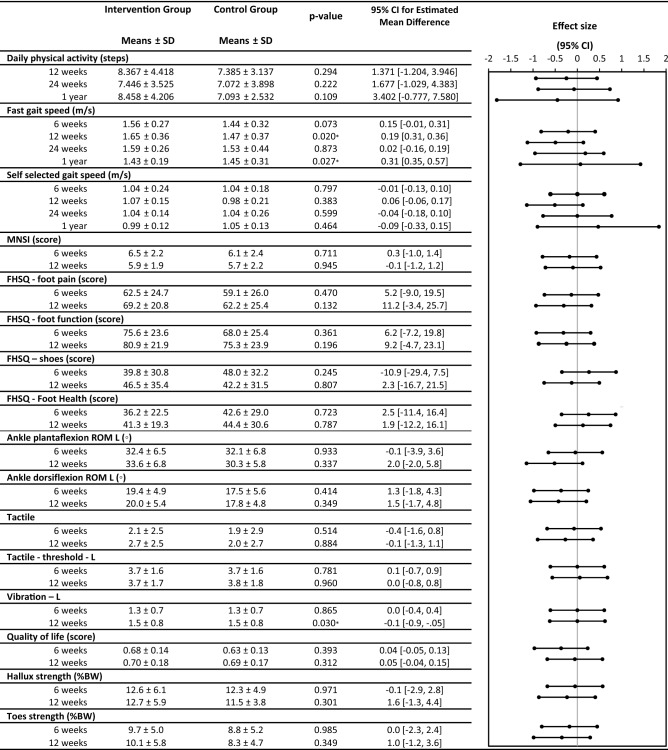
Secondary and primary outcomes from intervention group and control groups.

The original Supplementary Tables file is provided below.

The original Article and accompanying Supplementary Information file have been corrected.

## Supplementary Information

Supplementary Tables.

