# Supplementary material for: Foot–ankle therapeutic exercise program can improve gait speed in people with diabetic neuropathy: a randomized controlled trial
Source: Sci Rep. 2022 May 9;12:7561. doi: 10.1038/s41598-022-11745-0 (PMC9082985; doi:10.1038/s41598-022-11745-0)
Supplement: Supplementary file 1 — Supplementary Tables. [file 41598_2022_11745_MOESM1_ESM.docx]

|  | **Intervention Group** | | **Control Group** | | **P-value** | **95% CI for estimated means difference** | **Effect size (95% CI)**  **(95% CI)** |
| --- | --- | --- | --- | --- | --- | --- | --- |
|  | **Estimated Means (SE)** | | **Estimated Means (SE)** | |  |  |  |
| **MNSI (score)** |  |  | | |  | |  |
| 24 weeks | 5.7 (0.4) | | | 6.4 (0.4) | 0.331 | -0.7 (-2.0 to 0.6) |  |
| 1 year | 5.3 (0.4) | | | 6.5 (0.4) | 0.086 | -1.2 (-2.5 to 0.1) |  |
| **FHSQ - FOOT PAIN (score)** | | | | | | |  |
| 24 weeks | 71.6 (5.4) | | 59.0 (5.7) | | 0.119 | 12.6 (-3.2 to 28.3) |  |
| 1 year | 62.9 (5.5) | | 60.5 (5.5) | | 0.760 | 2.4 (-13.3 to 18.2) |  |
| **FHSQ - FOOT FUNCTION (score)** | | | | | | |  |
| 24 weeks | 79.4 (5.0) | | 73.8 (5.8) | | 0.465 | 5.6 (-9.4 to 20.6) |  |
| 1 year | 79.2 (5.2) | | 75.7 (5.7) | | 0.647 | 3.5 (-11.6 to 18.6) |  |
| **FHSQ - SHOES (score)** | | | | | | |  |
| 24 weeks | 53.0 (6.7) | | 35.9 (7.9) | | 0.105 | 17.1 (-3.6 to 37.8) |  |
| 1 year | 50.9 (7.0) | | 48.7 (7.8) | | 0.836 | 2.2 (-18.7 to 23.1) |  |
| **FHSQ - FOOT HEALTH (score)** | | | | | | |  |
| 24 weeks | 35.5 (8.3) | | 46.0 (8.5) | | 0.177 | -10.5 (-25.7 to 4.7) |  |
| 1 year | 37.5 (8.4) | | 51.2 (8.4) | | 0.079 | -13.7 (-28.9 to 1.5) |  |
| **ANKLE PLANTAFLEXION ROM R (**◦**)** | | | | | | |  |
| 6 weeks | 29.6 (1.3) | | 33.0 (1.6) | | 0.114 | -3.4 (-7.6 to 0.8) |  |
| 12 weeks | 30.3 (1.4) | | 33.0 (1.6) | | 0.233 | -2.7 (6.9 to 1.7) |  |
| 24 weeks | 32.5 (1.5) | | 30.9 (1.9) | | 0.543 | 1.6 (-3.4 to 6.5) |  |
| 1 year | 26.6 (3.3) | | 27.8 (2.2) | | 0.767 | -1.2 (-9.1 to 6.7) |  |
| **ANKLE PLANTAFLEXION ROM L (**◦**)** | | | | | | |  |
| 24 weeks | 34.9 (1.4) | | 31.4 (1.7) | | 0.135 | 3.5 (-1.1 to 8.1) |  |
| 1 year | 29.6 (3.1) | | 27.1 (2.2) | | 0.514 | 2.5 (-5.0 to 10.1) |  |
| **ANKLE DORSIFLEXION ROM R (**◦**)** | | | | | | |  |
| 6 weeks | 18.0 (1.2) | | 15.6 (1.4) | | 0.158 | 2.4 (-0.9 to 5.7) |  |
| 12 weeks | 20.2 (1.2) | | 16.6 (1.4) | | 0.048* | 3.6 (0.3 to 7.1) |  |
| 24 weeks | 17.0 (1.4) | | 16.6 (1.7) | | 0.872 | 0.4 (-3.8 to 4.4) |  |
| 1 year | 20.0 (3.0) | | 20.0 (2.1) | | 0.988 | 0.0 (-7.3 to 7.3) |  |
| **ANKLE DORSIFLEXION ROM L (**◦**)** | | | | | | |  |
| 24 weeks | 17.5 (2.2) | | 18.2 (2.4) | | 0.710 | -0.7 (-4.6 to 3.1) |  |
| 1 year | 18.2 (3.3) | | 21.1 (2.6) | | 0.399 | -2.9 (-9.6 to 3.8) |  |
| **TACTILE** | | | | | | |  |
| 24 weeks | 1.3 (0.4) | | 2.3 (0.5) | | 0.126 | -1.0 (-2.4 to 0.3) |  |
| 1 year | 0.9 (0.7) | | 1.8 (0.6) | | 0.366 | 0.1 (-2.9 to 1.0) |  |
| **TACTILE - THRESHOLD – R** | | | | | | |  |
| 6 weeks | 4.0 (0.2) | | 3.7 (0.3) | | 0.465 | 0.3 (-0.5 to 1.1) |  |
| 12 weeks | 3.8 (0.2) | | 3.8 (0.3) | | 0.911 | 0.0 (-0.9 to 0.9) |  |
| 24 weeks | 3.8 (0.3) | | 4.4 (0.3) | | 0.256 | -0.6 (-1.5 to 0.4) |  |
| 1 year | 3.2 (0.6) | | 3.6 (0.4) | | 0.595 | -0.4 (-1.9 to 1.1) |  |
| **TACTILE - THRESHOLD - L** | | | | | | |  |
| 24 weeks | 3.5 (0.3) | | 4.4 (0.3) | | 0.073 | -0.9 (-1.8 to 0.08) |  |
| 1 year | 3.7 (0.5) | | 4.3 (0.4) | | 0.402 | -0.6 (-2.0 to 0.8) |  |
| **VIBRATION – R** | | | | | | |  |
| 6 weeks | 1.4 (0.1) | | 1.5 (0.1) | | 0.677 | -0.1 (-0.5 to 0.3) |  |
| 12 weeks | 1.5 (0.1) | | 1.4 (0.1) | | 0.758 | 0.1 (-0.3 to 0.5) |  |
| 24 weeks | 1.7 (0.1) | | 1.2 (0.2) | | 0.061 | 0.5 (-0.02 to 1.0) |  |
| 1 year | 1.5 (0.3) | | 1.9 (0.2) | | 0.312 | -0.4 (-1.2 to 0.4) |  |
| **VIBRATION - L** | | | | | | |  |
| 24 weeks | 1.4 (0.1) | | 1.3 (0.2) | | 0.910 | 0.1 (-0.5 to 0.5) |  |
| 1 year | 1.1 (0.3) | | 2.1 (0.2) | | 0.023* | -1.0 (-1.8 to -0.1) |  |
| **QUALITY OF LIFE (score)** | | | | | | |  |
| 24 weeks | 0.71 (0.03) | | 0.60 (0.04) | | 0.048* | 0.11 (0.001 to 0.21) |  |
| 1 year | 0.68 (0.03) | | 0.69 (0.03) | | 0.886 | -0.01 (-0.11 to 0.09) |  |
| **HALLUX STRENGTH (%BW)** | | | | | | |  |
| 24 weeks | 12.0 (1.1) | | 12.7 (1.2) | | 0.669 | -0.7 (-4.1 to 2.6) |  |
| 1 year | 12.5 (1.8) | | 10.9 (1.5) | | 0.512 | 1.6 (-3.1 to 6.3) |  |
| **TOES STRENGTH (%BW)** | | | | | | |  |
| 24 weeks | 8.2 (0.8) | | 8.3 (1.1) | | 0.983 | -0.1 (-2.8 to 2.7) |  |
| 1 year | 9.2 (1.5) | | 7.0 (1.1) | | 0.256 | 2.2 (-1.6 to 6.0) |  |

**Supplementary Material**

**Table 1:** Secondary outcomes results from the intervention group and control groups in the 24-week and 1-year followup.

**Table 2:** Foot-ankle therapeutic exercise program description.

| **Categories** |  | **Exercise** | **Progression** |
| --- | --- | --- | --- |
| **WARMING EXERCISES** | Level 1 | 1. Stretching of the sole of the foot | 2x 30 sec → 2 x 1 min → 3 x 1 min |
|  |  | 2. Massage with the ball | 1 x 1 min → 2 x 1 min → 3 x 1 min |
|  |  | 3. Move your feet up, down and in circles | 1 x 10 rep → 2 x 10 rep → 3 x 10 rep → 4 x 10 rep |
|  |  | 4. Calf muscle stretching | 1 x 20 sec |
|  | Level 2 | 5. Writting words with your feet | short words → long words |
|  |  | 6. Interlacing your fingers and toes and making circular movements | 1 x 20 rep each foot |
|  |  | 7. Toes Manipulation | 1 x 20 rep each toe |
|  |  | 8. Massage with the ball without contact of the heel | press during 1 min each foot |
|  |  | 9. Self-massage | 1 x 20 sec |
| **INTRINSIC MUSCLES EXERCISES** | Level 1 | 1. Toe alternate | 1 x 10 rep (sitting)→ 1 x 10 rep (standing) |
|  |  | 2. Pick up objects with your toes | Cotton 1 x 10 rep; 2 x 10 rep. Ball 1 x 10 rep; 2 x 10 rep. Pencil 1 x 10 rep; 2 x 10 rep. |
|  |  | 3. Wringing towel with feet | 1 x 5 rep → 1 x 10 rep |
|  |  | 4. Open and close the toes (from the second to the fifth) | 1 x 10 rep → 1 x 20 rep → 1x 10 rep (with elastic bands) → 1 x 20 rep (with elastic bands) |
|  |  | 5. Squeeze toes separators | 1 x 10 rep → 2 x 10 rep → 3 x 10 rep (each foot) |
|  | Level 2 | 6. Toes flex with theraband | 1 x 10 rep → 2 x 10 rep → 2 x 10 rep (standing) |
|  |  | 7. Plantar arch raise | 1 x 10 rep → 2 x 10 rep → 3 x 10 rep |
|  |  | 8. Walk with your toes pressed to the floor | 1 x 10 steps, pressing 1 sec → 2 x 10 steps → 3 x 10 steps |
|  |  | 9. Short-foot exercise | 1 x 10 rep → 2 x 10 rep → 3 x 10 rep |
| **ANKLE EXERCISES** | Level 1 | 1. Climb on the tip Feet | 1 x 5 rep → 1 x 10 rep → 1 x 15 rep |
|  |  | 2. Kick the floor | 1 x 30 rep → 2 x 30 rep → 2 x 40 rep |
|  |  | 3. Tighten the ball | 1 x 10 rep → 1 x 15 rep → 1 x 20 rep |
|  |  | 4. Step forward and backward | 2 x 15 rep → 2 x 20 rep → 2 x 30 rep |
|  | Level 2 | 5. One Foot Balance | 1 x 10 sec → 2 x 10 sec → 1 x 20 sec → 2 x 20 sec |
|  |  | 6. Strengthening the medial musculature of the foot | 1x10 rep (yellow elastic band) → 1x10 rep (red elastic band) |
|  |  | 7. Strengthening the lateral musculature of the foot | 1x10 rep (yellow elastic band) → 1x10 rep (red elastic band) |
| **FUNCTIONAL EXERCISES** | Level 1 | 1. Walking across the steps | 1 x 5 rep → 1 x 10 rep → 1 x 15 rep |
|  |  | 2. Walk changing direction | 1 x 10 rep |
|  |  | 3. Walk changing direction (Diagonal) | 1 x 10 rep |
|  | Level 2 | 4. Mwalk through obstacles | 2 x 15 rep → 2 x 20 rep → 2 x 30 rep |
|  |  | 5. Walking through obstacles on unstable ground. | 1 x 10 rep → 1 x 15 rep → 1 x 20 rep |
